# A qualitative study of young workers’ experience of the psychosocial work environment and how this affects their mental health

**DOI:** 10.1186/s12889-024-20760-x

**Published:** 2024-11-29

**Authors:** Malte van Veen, Roosmarijn MC Schelvis, Paulien M Bongers, Karen M Oude Hengel, Cécile RL Boot

**Affiliations:** 1 Netherlands Organisation for Applied Scientific Research TNO, Unit Healthy Living & Work, Sylviusweg 71, Leiden, 2333 BE The Netherlands; 2 Amsterdam UMC location Vrije Universiteit Amsterdam, Public and Occupational Health, Boelelaan, Amsterdam, 1117 The Netherlands; 3 Amsterdam Public Health, Societal Participation & Health, Amsterdam, The Netherlands; 4 Body@Work, Research Center on Work, Health and Technology, TNO/Amsterdam Umc, Amsterdam, The Netherlands; 5 Public and Occupational Health, Amsterdam UMC, location University of Amsterdam, Meibergdreef 9, Amsterdam, The Netherlands

**Keywords:** Mental health, Young workers, Psychosocial work factors, Interview study

## Abstract

**Background:**

The evidence base for the relationship between psychosocial work factors and mental health focuses primarily on the general working population but little is known about young workers. The aim of this qualitative study is to identify psychosocial work factors that affect the mental health of young workers, with a focus on (1) *novel factors* of the psychosocial work environment that are relevant for young workers but have not been described in the literature and (2) experiences of psychosocial work factors associated with mental health that are specific to and typical for young workers.

**Methods:**

Semi-structured interviews were held with 36 workers aged up to 30. Participants were asked to describe work situations that affected their mental health. Factors were identified using a combination of inductive and deductive coding and open-coded factors were mapped onto the Copenhagen Psychosocial Questionnaire (COPSOQ), which is widely used as a framework for psychosocial work factors.

**Results:**

Most of the psychosocial factors mentioned by the young workers could be mapped onto the COPSOQ framework and were therefore similar to the general working population. Novel factors identified by this study were “*Procedural support*” and “*Responsibility for others*”. We also identified young-worker-specific experiences of psychosocial work factors associated with mental health (i.e. *Quantitative Demands*,* Influence at Work*,* Commitment to the Workplace*,* Job Insecurity*,* Quality of work*,* Job satisfaction*, and *Vertical Trust*). Lastly, young workers did not report the COPSOQ factor *Insecurity over working conditions* and *Work-life conflict* was reported as an indicator of mental health status rather than being perceived as a factor of the psychosocial work environment.

**Conclusions:**

Psychosocial work factors and their influence on mental health reported by young workers in this qualitative study are comparable to what is reported for the general working population. There are however some young-worker-specific experiences of psychosocial work factors and two novel factors. The novel factors, “*Procedural support”* and “*Responsibility for others”* are not found in common psychosocial work factor frameworks and might be studied specifically in relation to young workers. Our results provide organisations with levers that can be used to create a psychosocial work environment that benefits the mental health of young workers.

**Supplementary Information:**

The online version contains supplementary material available at 10.1186/s12889-024-20760-x.

## Background

Most young workers (aged ≤ 30) starting their professional career find that work contributes positively to their mental health [1]. Yet poor psychosocial work circumstances can have an adverse effect on mental health [2, 3]. In particular, the experience of poor psychosocial working conditions on entering the job market has been shown to lead to worsening mental health for young workers [4]. Psychosocial work factors found to be associated with mental health among the general working population are high job demands, effort-reward imbalance, job insecurity, and low organisational justice [5]. However, systematic reviews focusing on young workers have shown that the evidence base on how and which psychosocial work factors affect the mental health of this population is weak, mainly due to the limited number of studies on this topic [6, 7].

Since it is open to question whether, and how, the findings from the general working population are applicable to young workers, it is important to study young workers as a distinct group. It is hypothesised that first jobbers and young workers in general have certain work-related needs that affect their mental health and that these needs are distinct from those of their older colleagues. In an overview article, Zacher and Froidevaux (2021) [8] present data from a systematic review, a meta-analysis and an original study respectively to suggest that younger workers are less capable of regulating their emotions than older workers [9], less committed to organisations [10] and have higher intentions to leave [11] ; however the authors also acknowledge that much remains unknown. Taris et al. (1992) [12] suggest that the mental health of young workers in particular is affected by having to juggle loyalties to different people at work and that young workers have more need for clear procedures in their jobs than their older colleagues. Ebner et al. (2006) [13] found that younger workers’ personal goal orientation is more focused on promotion (i.e. motivation to achieve gains), whereas older workers’ personal goal orientation is more focused on prevention (i.e. motivation to avoid losses). This may lead to age-related differences on the work floor in terms of how young workers act and appraise the psychosocial work environment compared to older workers. However, a recent systematic review we performed acknowledged that much is still unknown about psychosocial work factors that affect the mental health of young workers given the high uncertainty of the evidence due to fuzziness in the conceptualization of outcomes and high study heterogeneity [7].

The assessment of how the psychosocial work environment affects mental health in ways that are typical for young workers is potentially limited because studies on the topic are mainly based on the existing traditional occupational health models (i.e. job demand-control-resource model [14] and effort-reward imbalance model [15]), leaving little room to identify novel factors and consider age-related particularities. As so little is known about the work-related mental health of young workers, research is needed to examine whether these existing models capture all aspects of the psychosocial work environment that are relevant for young workers. A primarily inductive, qualitative exploratory study not guided by existing models, with a targeted sample of young workers, allows for the identification of previously overlooked psychosocial work factors. Such an approach also allows for the identification of known psychosocial work factors associated with mental health that are perceived and experienced in ways that are specific to and typical for young workers.

Against the background described above, the aim of the current study is to identify psychosocial work factors that affect mental health of young workers, with a focus on (1) *novel factors* of the psychosocial work environment that are relevant for young workers but have not been described in the literature and (2) young-worker-specific experiences of psychosocial work factors that are well-described in the literature.

## Methods

### Study design

We applied a qualitative research design, using semi-structured interviews. All interviews were conducted online via Microsoft Teams. Since we are interested in the experiences of young workers, rather than providing them with a definition that would be used throughout the interview, we asked the study participants to define mental health in their own words. Specifically, they were asked to describe experiences, emotions, feelings, behaviours, and mental states they associate with their personal mental health. We used this description throughout the interview. Following the suggestions by Braun and Clarke (2006) [16] for conducting thematic analysis, we attempt to make explicit the epistemological underpinnings of our study: we believe that the psychosocial work circumstances that the young workers are describing are commonly experienced by young adults in Dutch workplaces. We adhere to COREQ Reporting Guidelines [17] (the COREQ checklist can be found in Additional file 1). By using these guidelines we also attempt to make explicit issues of reflexivity (i.e. the way our own personal biases may have affected the results).

### Participant selection and recruitment

We employed purposive sampling based on the criteria of *sex* (male versus female), *educational level* (vocational education versus (at least) a college degree), and whether the young worker had an *interpersonal or non-interpersonal job*. We used these sampling criteria in light of research indicating that mental health at work differs according to sex [18], across educational groups [19], and by type of work [20]. Young workers only qualified for inclusion if they were between 18 and 30 years old and worked at least 16 h per week. Eligibility for inclusion was double-checked at the beginning of each interview.

Participants were recruited through the social media platforms LinkedIn and Instagram with a poster and a video in which author MVV asked young workers to participate. The team of authors reposted the message on their LinkedIn profiles to increase the reach. Additionally, we approached young worker and young adult advocacy groups, some of which shared our recruitment request on their own channels and platforms. Interested participants could sign up for the study by clicking a link in the poster and social media posts. This led them to a landing page where they completed an application form, noting their age, sex, educational level, role and job title. They were then redirected to an online scheduling tool where they could choose a date and time for the interview from the time slots offered by the researchers. As a token of appreciation for their participation, the participants received a €25 online gift card. Participants were categorised as having an interpersonal or non-interpersonal job based on their responses. This categorisation into type of work was done by author MVV.

The online recruitment procedure was successful and quickly yielded a large number of applications for the interviews. However, the fast pace of applications and scheduled interviews made it difficult for the researchers to adhere to the specified purposive sampling criteria and highly educated females were overrepresented in the initial sample. Therefore, in a second recruitment request issued through the same online channels, we specifically asked males and persons with vocational-level education to apply for an interview.

Ultimately, the study population consisted of 36 young workers: 29 females and seven males. Eight had completed vocational education and 28 had at least a college degree. Of the 36 interviewees, 15 were working in interpersonal jobs and 21 in non-interpersonal jobs. Males with a college degree and a non-interpersonal job were not represented in the final sample. Non-response was limited: one participant did not show up for a scheduled interview for reasons unknown and did not respond to subsequent contact attempts.

### Data collection

Semi-structured interviews were held between April and June of 2023. The first set of interview questions concerned the interviewee’s past and current work life. This was followed by a set of questions on the interviewee’s mental health in general. In the central section of the interview, interviewees were asked to describe situations at work that affected their mental health in some way. These situations were then explored with loosely structured follow-up questions to identify the psychosocial work factors involved. This meant that all results concerning the psychosocial work environment were discussed in the context of their impact on personal mental health. The interview protocol, which can be found in Additional file 2, was piloted by researchers interviewing each other (RS interviewing MVV and MVV interviewing LA).

Participants were asked to choose a setting where they could be interviewed without interruption. The interviews lasted between 40 and 60 min. All but two interviews were conducted in Dutch, which was the native language of those interviewed in Dutch. None of the individuals involved in the two interviews conducted in English spoke English as a native language. All interviews were conducted by two researchers in varying roles, with MVV being present at all interviews. One researcher led the interview, the other took notes and asked follow-up questions if they felt it would be informative.

A summary of the interview based on the notes taken by the second researcher was shared with the participant for confirmation and possible amendments. Audio recordings of the interviews were made and transcribed by an external party.

At the beginning of the interview the interviewers briefly introduced themselves by name and explained their professional background and the context in which the study was being conducted, i.e. as part of MVV’s doctoral research. After the interview, we sent the participant a summary of the interview to check that we had understood them correctly. We also asked for feedback on the interview. This did not yield any feedback that required us to make changes to the interview protocol.

### Interviewer characteristics

Author MVV, MSc, 32-year-old male, background in social psychology and epidemiology, was present at all interviews. Three interviews were conducted with author RS, PhD, 37-year-old female, 32 interviews were conducted with JM, MSc, 25-year-old female, and one interview was conducted with LA, MSc 28-year-old female (for the latter two interviewers see the Acknowledgements). All interviewers were employed by the Amsterdam UMC Department of Public and Occupational Health at the time of data collection. All interviewers were experienced in conducting interviews.

### Data analysis

**Fig. 1:**
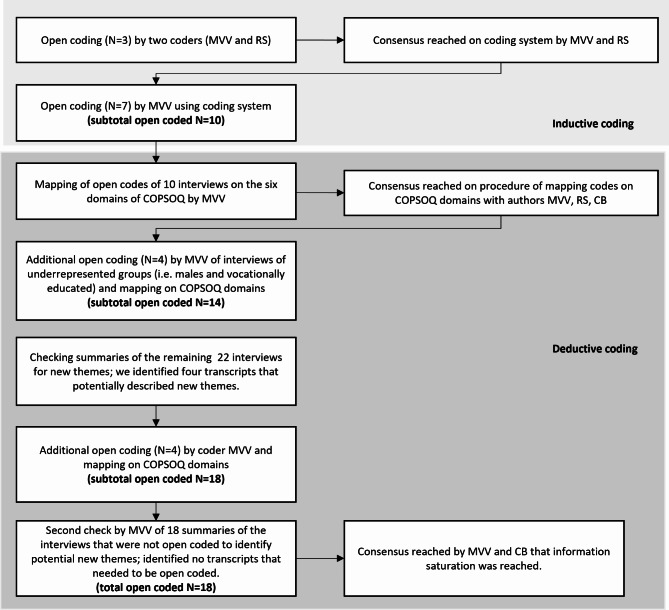
Flowchart of the coding proces. Initial inductive coding was based on ten interviews, followed by a deductive process of mapping open codes on the six COPSOQ domains as well as additional open coding of interviews until information saturation was reached. This resulted in 18 interviews being coded in total

We combined an inductive and deductive approach, giving primacy to the inductive components (see Fig. 1. Flowchart of coding process). Data analysis began after all interviews had been conducted. Coding was done using MAXQDA software. In the inductive phase, MVV and RS open-coded the first three interviews and reached a consensus on further open coding. MVV then open-coded another seven interviews. In the deductive phase, the codes of these ten interviews were grouped, using the third version of the Copenhagen Psychosocial Questionnaire COPSOQ [21] as a framework, and discussed by three of the authors (MVV, RS, CB) in a consensus meeting. The framework was thought to encompass the most common and important psychosocial work factors for the general working population divided into eight domains, of which our study included the first six, namely *Demands at Work*, *Work Organisation and Job Content*, *Interpersonal Relations and Leadership*, *Work-Individual Interface*, *Social Capital*, and *Conflicts and Offensive Behaviour*. We did not include the last two domains *Health and well-being* and *Personality* as they do not characterise the psychosocial work environment. Where open codes could not be mapped onto the COPSOQ reference framework, they constituted a novel (not previously described) psychosocial work factor perceived and appraised by young workers. Young-worker-specific experiences of COPSOQ factors plus potential novel factors constitute the main themes of the analysis. With the aim of analysing information from different groups of young workers, another four interviews were open-coded in order to achieve a more equal distribution of the background characteristics on which the purposive sampling strategy was based. With these 14 open-coded interviews serving as a basis, the summaries of all remaining interviews were checked for potential new themes. This led to the open-coding of another four interviews, such that 18 of the interview transcripts were open-coded and grouped using the COPSOQ framework. The summaries of the other 18 interviews were rechecked by author MVV for potential new topics that may have revealed new themes. This check involved reading the summary with all identified themes in mind and noting any new themes that appeared. When no new themes appeared, authors MVV and CB agreed that data saturation had been reached.

The identified themes (i.e. psychosocial work factors) were then grouped into four categories: (1) experience of the psychosocial work factor is more specific to and typical for young workers in terms of its relationship to mental health, (2) factor appears to be comparable to the general working population and not typical for young workers in terms of its relationship to mental health, (3) factor is not covered by COPSOQ, and (4) factor is not mentioned by young workers. We considered the experience of a factor to be more specific to and typical for young workers (category 1), when age, life course and/or early career-related aspects were included in the way the factor was described by the young worker. We used COPSOQ guidelines and the author team’s knowledge of the literature to make the comparison with the general working population (category 2) and discussed this in a consensus meeting (MVV, RS, CB) and after each draft of the manuscript.

We present quotes to support our analysis of young worker experiences of psychosocial work factors (category 1) and novel factors (category 3), selecting one or two quotes that are most illustrative of the point we want to make. In the case of the psychosocial work factors that are comparable to the general working population (category 2), we only present a quote if this adds to the understanding of the psychosocial work factor.

## Results

### Mental health

Young workers associate good personal mental health with having enough energy to spend their days the way they want, including work and leisure activities. Indicators of poor mental health are feeling exhausted and irritable, and lacking the energy to engage in social activities after work. Young workers also mentioned “not feeling oneself” as an indicator of poor mental health.

[when thinking about good mental health] I mainly think: getting through the day with ease. And fortunately, if you are mentally healthy then you are happy and life comes easy to you. That’s what I would classify as that. Well let’s say things are going well, do I have a lot of energy? Do I have a lot of headspace to meet people and do new things? Those kind of things. And suppose my mental health was lower (sic.), then I don’t have those things. So then I have less energy, I am more introverted. Then life just looks less rosy.

Interviewee #9, college-educated female in a non-interpersonal job

“I think a good work-life balance, partly, in terms of energy and such. Mental health means that you yourself… Yes, I really think: finding a good balance. And that you are really enjoying yourself and make yourself comfortable. Also in private life, but also at work, in this case. That you find your challenges. That you continue to develop. To me, that is my mental health”.

Interviewee #36, vocational-level educated male in a non-interpersonal job

### Psychosocial work environment

Per COPSOQ domain, we discuss the psychosocial work factors, and combinations of psychosocial work factors, that affect mental health in a way that is typical for young workers. The category system used can be found in Additional file 3. We also discuss novel factors and factors that appeared similar to what we know from the literature on the general working population (see Table 1 for an overview).

**Table 1:** Overview of psychosocial work factors per domain, categorised as typical for young workers, similar to the general working population, novel with regard to the COPSOQ and not mentioned

Psychosocial Work Factor Domain	Psychosocial work factor experiences and appraisals more typical for young workers	Psychosocial work factors experienced by the general working population	Novel psychosocial work factors not covered by the COPSOQ	Psychosocial work factors not mentioned
A Demands at Work	Quantitative demands	Work pace, Cognitive demands, Emotional demands, Demand for hiding emotions	Responsibility for others	NA
B Work Organisation and Job Content	Influence at work	Possibilities for development, Variation of work, Control over working time, Meaning of work	None	NA
C Interpersonal Relations and Leadership	None	Recognition, Role clarity, Illegitimate tasks, Quality of leadership, Social support from colleagues, Social support from supervisors, Sense of community at work, Predictability	Procedural Support	NA
D Work-Individual Interface	Commitment to the workplace, Job insecurity, Quality of work, Job satisfaction	Work engagement	None	Insecurity over working conditions, Work-life conflict
E Social Capital	Vertical trust	Horizontal trust^1^, Organisational justice	None	NA
F Conflicts and Offensive Behaviour	None	Harassment and gender-based discrimination	None	NA

^1^ Horizontal trust is conceptually closely related to the Domain C COPSOQ factor *Sense of community at work*. The distinctive sign of a lack of horizontal trust as defined by the COPSOQ, namely “withholding information” from colleagues or management, was not reported by young workers

NA = Not applicable

### Domain A: demands at work

Young workers described all but one of the psychosocial work factors in this domain in ways that appeared to be similar to the COPSOQ description for the general working population. Only the experience of *Quantitative demands* was typical for young workers. Additionally, young workers described the novel factor “*Responsibility for others*” (Table 1).

**Experiences more typical for young workers.** Concerning *Quantitative demands*, young workers said that having too little work was undesirable and being told that this was normal during an onboarding period did not help. At the same time young workers also mentioned the risk of creating a problematically high workload for themselves when being asked to do tasks because of the difficulty of saying no. Young workers said it was sometimes difficult to reject work because they felt they had to prove themselves.

If someone says, do this and this, then I will not immediately say in a new position, ‘no, I am not going to do this’, or ‘I don’t think that is how it should be done’. Then I’ll accept it anyway.

Interviewee #27, college-educated male in an interpersonal job

That is really something of which I thought: when I have put this together, then I really have something nice to put in my track record. Very cool in the beginning, [it gave] a lot of energy. It also took up a lot of my time. But I had to do that in consultation with my boss. My boss and the boss of [other organisation] we worked with continuously disagreed […] At a certain point we made a call ourselves […]. My boss ultimately did not agree with that although I had run it by him, maybe his thoughts were elsewhere. So that was very confusing for me. And that particular day that we went live, I had my first panic attack.

Interviewee #1, college-educated female in an interpersonal job

**Experiences similar to the general working population.** *Work pace*,* Cognitive demands*,* Emotional demands*, and *Demand for hiding emotions* were all mentioned by young workers but were not perceived in ways that appeared to be more typical for young workers than for the general working population.

**Novel factor: Responsibility for others.** A factor mentioned by young workers but not covered by the COPSOQ framework was “*Responsibility for others*”. Having a role that meant that one’s work might negatively affect others (such as colleagues, patients, students, and interns) was sometimes perceived as a burden for young workers.

I had the feeling that without me nothing would happen and that was the case indeed. If I didn’t go to work, people wouldn’t have an internship.

Interviewee #4, college-educated male in an interpersonal job

I felt a great responsibility towards the students. Like: okay, then there will be nothing prepared for them [the students]. I want that there is something prepared for them.

Interviewee #8, college-educated female in an interpersonal job

### Domain B: work organisation and job content

Young workers’ association of mental health with psychosocial work factors belonging to the domain of *Work Organisation and Job Content* appears to be mostly similar to the COPSOQ description for general working population. Only the experience of *Influence at work* as a psychosocial work factor was specific to and typical for young workers (Table 1).

**Experience more typical for young workers.** Concerning *Influence at work*, young workers said they wanted to have influence on the way they carry out tasks at work, particularly when they have developed the necessary skills through education or earlier work experience. This was less important to them when doing tasks for which they felt they lacked competence. Young workers also said that they did not raise issues at work because they felt too junior to object.

I remember, I had just graduated and had two master’s degrees […] and had skills and then I had to crop images in photoshop and pick up the mail. [When I said something about it] I received feedback that I should first take a closer look at how an organisation was put together before I started doing my own things.

Interviewee #22, college-educated female in a non-interpersonal job

“At my previous job I had to figure out everything myself, how everything worked and how it all… While I was actually not trained for that, not sufficiently trained for it.”

Interviewee #4, college-educated male in an interpersonal job

**Experiences similar to the general working population.***Possibilities for development*, *Variation of work*, *Control over working time*, and *Meaning of work* were all mentioned by young workers but were not perceived in ways that appeared to be more typical for young workers than for the general working population.

### Domain C: interpersonal relations and leadership

We did not find any experiences that were specific to or more typical for young workers. All factors were similar to the COPSOQ description for general working population. These factors are *Recognition*, *Role clarity*, *Illegitimate tasks*, *Quality of leadership*, *Social support from colleagues*, *Social support from supervisors*, *Sense of community at* work, and *Predictability*. In addition, we identified a novel factor that we labelled “*Procedural support*” (Table 1).

**Novel factor.** A factor described by young workers but not covered by the COPSOQ was “*Procedural support”*. Young workers expressed a need to be presented with clear procedures and a desire for established ways of doing things within an organisation. While young workers felt it was important to have influence on how they carried out their tasks, they appreciated having established structures to fall back on.

Yes, I think there is clarity in the state of affairs, that if you are busy carrying out your work, that you know if A happens, then I can go to B to get C done, so to speak, that you just know where you stand, I need that in any case.

Interviewee #4, college-educated male in an interpersonal job

I enter a company and you just notice: the systems are not set up, things are shared via WhatsApp groups. Declarations… how the declarations are shared via WhatsApp groups. So I can already see a bit of: this is an up and coming company, while, it was not even really an up and coming company.

Interviewee #33, vocational-level educated female in a non-interpersonal job

### Domain D: work-individual interface

All but one of the psychosocial work factors in this COPSOQ domain were associated with mental health in ways that were typical for young workers. The only factor not perceived in a way that was specific to young workers was *Work engagement*. No novel factors were identified (Table 1).

**Experiences more typical for young workers.** To feel *Commitment to the workplace*, young workers had to experience their work as meaningful, i.e. their employer had to contribute to a societally relevant goal they identified with to secure their commitment to the organisation. One young worker’s impression was that commitment to the workplace increases with tenure. Young workers also explicitly stated that not being too committed to one’s workplace could be a healthy buffer against exceeding one’s limits in a way that would negatively affect mental health.

Money is nice, but I deliberately chose this job and this employer, because it is a socially very relevant topic, like the energy transition. There’s just a lot happening and it affects everyone.

Interviewee #5, college-educated female in a non-interpersonal job

Concerning *Job insecurity*, having a temporary contract with an uncertain prospect of contract renewal was a significant stressor for young workers. Conversely, job security meant that young workers were less likely to exceed their limits and therefore less prone to mental health issues.

Of course I can also say: okay, I’m just going to work less hard, or I’ll just deliver less work, less good work, but of course I never wanted that up until now. I just wanted to perform well and show my best side. But I also think that because I have now been given that permanent position and somewhat more the guarantee that I’m good. I now dare to let go a little more. Like: okay, I’m not going to work the whole evening now, then they’ll just have to wait for that report.

Interviewee #8, college-educated female in an interpersonal job

Concerning *Quality of work*, experiencing a lack of competence to deliver work of sufficient quality was perceived as stressful. Another frustration related to quality of work was that more senior colleagues expressed an unwillingness to adapt their work even if that would lead to better results. Additionally, young workers said they could feel insecure about their own skills when comparing themselves to the older colleagues. Being aware that their work was sloppy due to poor mental health was seen as part of a vicious circle that increasingly undermined the quality of their work.

I noticed that I was underperforming and becoming sloppy and no longer paying close attention to the details. So those were signals for me, and especially if you do that… at some point you end up in a vicious circle. The situation makes you function less, but if it is also emphasised that you are not doing things right, you get even deeper into it.

Interviewee #2, college-educated male in an interpersonal job

Concerning the salary aspect of *Job satisfaction*, some young workers said satisfaction with their salary was sufficient for well-being, at least for a side job. Other young workers were inclined to see satisfaction with their salary as a necessary but not sufficient condition for well-being.

**Experience similar to the general working population.** Concerning *Work engagement*, young workers said they did not like to experience a lower level of work engagement since this led them to spend less time on work activities and adversely affected the quality of their work. In other words, they saw reduced work engagement as a consequence and not just a cause of worsened mental health. More specifically, a lower level of work engagement was described as a way of coping with poor mental health, particularly for those who aspired to do societally relevant work.

I just don’t really know what I could have done differently, except maybe trying my best a bit less. But I find that very difficult. […] Maybe also be more patient and distance myself a little more mentally or emotionally from my work. I could have done that, but I actually don’t want to.

Interviewee #32, college-educated female in an interpersonal job

Notably, *Insecurity over working conditions* was not mentioned by the young workers and *Work-life conflict* was described as an indicator of mental health status rather than being perceived as a factor of the psychosocial work environment.

### Domain E: social capital

In this domain only the factor of *Vertical trust* was perceived in a way that was typical for young workers. Their experiences of the factors of *Horizontal trust* and *Organisational justice* were similar to the COPSOQ description for the general working population (Table 1).

**Experience more typical for young workers.** Concerning *Vertical trust*, young workers said it was important that they did not feel controlled by their supervisor, since this made them feel insecure, especially if the young worker lacked confidence in their own competence. Young workers illustrated vertical trust as being able to do a ‘sanity check’ with a trusted supervisor in a disagreement with colleagues.

If I notice that my work is being monitored, from which I infer that the other person either wants to take over, or if I notice that the manager […] is having a kind of suspicious idea, like “did you actually do it like this”, or “did you actually do it in this particular way?” And I think it is important to be trusted in how I do it and what I do.

Interviewee #2, college-educated male in an interpersonal job

### Domain F: conflicts and offensive behaviour

Overall experiences associated with this domain were occasionally reported by young workers and described as having a detrimental effect on their mental health. Nevertheless, being a young worker was not reported to be a key factor in the experience (Table 1).

## Discussion

Young workers reported a broad set of mental health-related psychosocial work factors that are also described in the COPSOQ. There is therefore considerable similarity between young workers and the general working population. This implies that factors known to affect mental health in the general working population, such as *Emotional demands*, *Possibilities for development* and *Recognition* [2] also affect the mental health of young workers. Across the different COPSOQ domains, this similarity was particularly evident for factors in domains *C Interpersonal Relations and Leadership* and *F Conflicts and Offensive Behaviour*. However, some psychosocial work factors were perceived in ways that appeared to be specific to and typical for young workers (i.e. *Quantitative Demands (A)*,* Influence at Work (B)*,* Commitment to the workplace (D)*,* Job insecurity (D)*,* Quality of work (D)*,* Job satisfaction (D)* and *Vertical Trust (E)*). We found this to be the case particularly for factors in the COPSOQ domain *D Work-Individual Interface* and for one of the factors in domains *A Demands at Work*,* B Work Organisation and Job Content* and *E Social Capital*. Additionally, we identified two novel factors, which appear to be typical for young workers. We labelled these (1) “*Procedural support”* and (2) “*Responsibility for others”* internal and external to the organisation.

### Experiences of psychosocial factors specific to young workers

Some psychosocial factors were perceived in ways that were specific to young workers (*Quantitative Demands*,* Influence at Work*,* Commitment to the Workplace*,* Job Insecurity*,* Quality of work*,* Job satisfaction and Vertical Trust*). We offer two lines of reasoning as to why the experiences reported by the group of young workers differed from the known relationships between the psychosocial work environment and mental health for the general working population. The first line of reasoning focuses on the fact that many of the young workers were in their first career job or reported situations they encountered in their first career job (typically referred to by the participants as their first “real job” as opposed to a side job they had as a student). This meant that many of the young workers did not have a frame of reference for workplace norms and their first work experiences were their only point of reference for what they considered normal in working life. This is illustrated by young workers exceeding their personal limits in ways they now say they would no longer do. While evidence is scarce for effects of lacking experiences with workplace norms on mental health, young workers’ lack of confidence to address problems at work has been identified as a risk for physical safety at work [22] and the same vulnerability might thus cause issues concerning mental health. Also, young workers may find it more difficult to access organisational resources (e.g. asking for and securing help from colleagues) due to their lack of experience and lack of familiarity with a new workplace, which has been reported as a threat to physical safety at work [22]. This contributes to the identified need for procedural support and is also reflected in the young workers’ awareness of their responsibility for others internal and external to the organisation.

The second line of reasoning concerns age effects (affecting an individual), period effects (affecting the whole population), and cohort effects (affecting groups of individuals within the population) [23], that are not limited to the work domain but reflect broader individual and/or societal developments. Our study reflects the perspectives of young workers in the current era and these workers generally belong to the same cohort. A recent meta-analysis of “generational differences” showed mixed and limited scientific support for meaningful differences between generations in relation to a variety of outcomes [24]. This calls into question the relevance of the idea of “generational differences”. The idea of “generational differences”, which is frequently proposed as an explanation when considering the mental health of young workers, could be another label for what appear to be very robust cohort effects. However, it is notoriously difficult to disentangle these three different types of effects and our qualitative study does not provide the opportunity to contrast groups within our collected data (i.e. young workers versus older workers, workers of all ages today versus workers of all ages in the past, young workers today versus young workers in the past).

### Novel psychosocial work factors for young workers

The novel factor we have labelled “*Procedural support*” resembles what Taris et al. (1992) [12] called “job clarity*”*. We are thus not the first to suggest or identify this factor. Job clarity has been shown to be a determinant of work-related outcomes, albeit not in relation to mental health (e.g [25]). An additional reflection on the novel factor of “*Procedural support”* as we labelled it is appropriate here. Whereas in the COPSOQ “role clarity” refers to clarity regarding one’s goals, responsibilities and expectations, the novel factor of “*Procedural support*” identified by this study refers to clarity about the way things are done within the organisation. This clarity serves as a support tool for our study population, because it makes the organisation predictable and reliable in the way that it operates when the work becomes difficult or if one encounters problems. We see this as distinct from concepts such as procedural and organisational justice described in occupational science [26], because procedural support is about knowing how things are done and not whether the way things are done feels fair or just. This reasoning is supported by the finding that the young workers’ experience of *Organisational justice* (Domain E - *Social Capital*) was similar to the general working population.

As for the novel factor of “*Responsibility for others”* internal or external to the organization, our impression is that this factor has not yet been widely researched in the literature on work-related mental health. However, a review of specific stressors for general practitioner trainees names “the sudden assumption of responsibility for patient care” (p.11 [27]), as a stressor for these young workers. This increase in responsibility at work might generalise to other professions. Conceptually, it is important to note that this factor is not only about the perception or appraisal of the young worker. From the interviews it appeared to be an objective feature of a particular job involving responsibility for others (e.g. providing care, supervising interns, delivering lessons to students). There is some overlap with *Emotional demands* as a psychosocial work factor in the sense that *Responsibility for others* is regularly found in jobs characterised by high emotional demands [20], particularly when those for whom the young worker is responsible have the impression that their needs are not met. However, the factor of *Emotional demands* focuses on being confronted with other people’s feelings regardless of the formal responsibility one has for these people (see e.g. COPSOQ items and definition [21]).

### Methodological considerations

Some methodological considerations should be taken into account when assessing our results. Firstly, our recruitment strategy relied mainly on Instagram and LinkedIn, limiting access for groups that are possibly less active on these platforms (e.g. blue collar workers [28]). Furthermore, vocational-level educated young workers and men were underrepresented in our study population. This potentially reduces the transferability of our results to individuals from these underrepresented groups. Although we reached data saturation with no new themes appearing in the final interviews, we cannot rule out the possibility that our study may have missed insights regarding the groups that were underrepresented in the final sample. Measures taken to strengthen the methodology of our study included the double-coding procedure for the first round of interviews and consensus meetings after subsequent rounds of coding.

Secondly, the reader should take into account the fact that our study used the COPSOQ as a reference framework. The phrase ‘similar to the general working population’ implies that the finding is ‘similar to the COPSOQ’. Although the COPSOQ is comprehensive and widely used in occupational science, there are other measurement instruments we could have chosen as a point of reference (such as the Job Content Questionnaire [29]. Had we done so, this might have led to a slightly different analysis.

Thirdly, we chose to do just one interview with each participant. This might have limited familiarisation between interviewers and interviewees. A disadvantage of this approach is that it is less conducive to the sharing of sensitive personal experiences and might explain why participants barely mentioned factors in COPSOQ Domain *F Conflicts and Offensive behaviour.* Hence, we cannot rule out the possibility that our study failed to identify psychosocial work factors and potentially even novel factors in this domain that are perceived in ways that are typical for young workers. A different research design is needed to address this.

Finally, the variation *within* any study sample based on how old workers are (age), when they live (period), and when they were born (cohort) should not be overlooked. In fact, ignoring within-group heterogeneity is one of the main criticisms of the use of “generational differences” as a scientific explanation in general [30]. In our study population, we accounted for heterogeneity within the group of young workers by applying a purposive sampling strategy. However, in our results we did not see clear distinctions along the lines of the purposive sampling criteria of sex, educational level and type of job.

### Implications for research and public health

Our results make it clear that more specialised research on young workers is needed, since our study suggests that, despite a considerable overlap, not all findings for the general working population are applicable to young workers. In line with this, we recommend that instruments used to analyse the impact of the psychosocial work environment are complemented with measurements of *Procedural support* and *Responsibility for others* when young workers are among the study population. Above we outline the need for repeated data collection on workers of different age groups to disentangle age, period, and cohort effects. Furthermore, it became apparent that there is a reciprocal relationship between mental health and psychosocial work factors, as illustrated by the statement quoted above: “[when mental health is poor] life just looks less rosy”. This altered perspective can also be assumed to affect the appraisal of the psychosocial work environment. This potentially reciprocal relationship poses a well-known challenge for research on work-related mental health and is known as the “triviality trap” [31]; research designs that focus more explicitly on identifying causal relationships are proposed to tackle this issue [32].

In terms of public health, our study is relevant because young workers experience work-related psychosocial factors in jobs in an ageing society. In many Western countries there is a correlation between retirement age and life expectancy [33]. Young workers will have to work for longer than previous generations and good mental health will be an important enabling resource. Future research could specifically take into account the COPSOQ factors not mentioned by our study population to expand our understanding of these factors and prevent them from potentially undermining mental health.

### Implications for practice

We conclude that young workers experience many psychosocial work factors in ways that are similar to the general working population. This implies that young workers do not need to be treated as a separate group with distinct features across all psychosocial work dimensions. At the same time, our results show that creating a good psychosocial work environment for young workers requires specific deliberation by organisations. In particular, employers and supervisors should pay attention to the mental health of young workers whose jobs are characterised by high responsibility for others. The young worker assigned the responsibility should be equipped with resources to do the job well and supported by a supervisor. Lastly, employees need access to interventions that address any associated stress. While the provision of procedural support could play a crucial role in these situations, it should also be considered for jobs other than those that are characterised by high responsibility for others. Organisations should make sure that their onboarding and managerial processes effectively support young workers, so they feel secure about “how things are done”. Furthermore, organisations should be cautious in assuming that young workers appreciate a high degree of autonomy. Research showed that within-person increases of job autonomy led to an increase of emotional exhaustion [34], which is in line with our finding that young workers experienced an increase in job autonomy as detrimental to their mental health. Organisations, and supervisors in particular, should actively provide procedural support for young workers while also trusting the existing skills and knowledge that the young worker brings to the job.

When applying our results in the context of a work environment it is important to note that our current study did not try to rank order the impact of individual psychosocial work factors on young workers’ mental health. *Sense of community at work*, for example, was widely mentioned by the study participants but was not perceived in a way that was specific to and typical for young workers. This means that the results of our study are not meant to be interpreted as dictating which factors organisations should prioritise given limited resources. Rather, our results indicate what needs to be taken into account in order to create a psychosocial work environment that supports the mental health of young workers.

## Conclusion

Young workers reported a broad set of psychosocial work factors that are also described in the COPSOQ. There is therefore considerable overlap between young workers and the general working population in terms of which psychosocial work factors affect workers’ mental health. There are however some dynamics that are more typical for younger workers, particularly in COPSOQ domains *A Demands at work* and *D Work-Individual Interface*. In addition, two novel factors, i.e. *Procedural support* and *Responsibility for others*, which are not found in the commonly used psychosocial work factor frameworks, might be particularly relevant for young workers. Our results provide organisations with levers that can be used to create a positive psychosocial work environment for young workers. One would be to examine young workers’ experience more closely when seeking to maximise their influence on how they carry out their work while at the same time providing an environment characterised by supervisor trust and procedural support.

## Electronic supplementary material

Below is the link to the electronic supplementary material.

Supplementary Material 1

Supplementary Material 2

Supplementary Material 3

## Data Availability

The interview protocol for this study can be found in additional file 2 The transcripts of the interviews can be requested from the corresponding author for scientific purposes in accordance with the consent provided by the study participants.
