# Supplementary material for: A qualitative study of young workers’ experience of the psychosocial work environment and how this affects their mental health
Source: BMC Public Health. 2024 Nov 29;24:3341. doi: 10.1186/s12889-024-20760-x (PMC11607927; doi:10.1186/s12889-024-20760-x)
Supplement: Supplementary file 2 — Supplementary Material 2 [file 12889_2024_20760_MOESM2_ESM.docx]

**The association of psychosocial work factors and mental health in young workers: a qualitative interview study.**

Malte van Veen, Roosmarijn MC Schelvis, Paulien M Bongers, Karen M Oude Hengel, Cécile RL Boot

**Appendix B – Interview Protocol**

- Check that the informed consent has been signed.

- Check: is the setting suitable for an interview?

- Start the recording.

[Introduction]: This conversation will last a maximum of 60 minutes. If you need a break, let us know and we can stop for a moment. If you have any questions about anything, please ask. And if anything is unclear, please say so.

Are you in a setting where you feel comfortable to have this conversation?

1. What is your age? Do you have an education degree? And if so, what programmes or courses did you follow?
2. Did you complete your degree(s)?
3. How long have you been working? Have you generally worked since completing your education?
4. Can you describe your career so far? [Follow-up: Ask approximately how long the interviewee held a position and when.]
5. Follow up if unclear:
6. Sector in which the interviewee worked?
7. Career, motivation to change job?
8. Conditions in each workplace?
9. [If not already clear from the answers to question 3:] Please describe a typical working day in your current job.
10. How important is work in your life compared to other things you do?
11. [If the interviewee finds the question difficult:] Do you mainly work to earn money so you can do things outside of work? Or are there other ways in which your work is meaningful to you?

[Transition:] I now have an idea of what your working life has been like so far, your current work and the importance of work in your life. At this point I would like to switch to mental health.

1. What comes to mind when you think about your own mental health? [Use the wording used by the interviewee throughout the interview.]
2. [If the interviewee finds the question difficult:] What do you notice about your mental health?
3. How has your mental health been over the course of your work life? [Repeat the wording used by the interviewee in answer to question 6.]
4. How would you rate your mental health at the moment on a scale of 1 to 10?
5. [If not already clear:] What makes you give this rating?

[Transition to bring the interviewee’s attention back to the interview situation after this question:] Do you need a moment to come back to the here and now? Feel free to get up or have a drink and we'll continue when you're ready.

[When the interviewee is ready:]

We are now going to look at situations at work in which your mental health played a role or that affected your mental health.

1. Can you describe a situation at work that made you feel [Use the interviewee's own words about mental health]?
2. Ask questions using the STAR method and downward arrow technique [identify core beliefs and assumptions]
3. Situation - What factors were at play?
4. Task/responsibility/role - Formal and felt responsibility in the situation?
5. Result - What was it about the situation that affected your mental health?
6. Antecedent - How did you approach the situation? Is there a history/context?
7. Development over time - How has this continued to influence your work?
8. What would it have taken to get a different result?
9. How could things have gone differently? How could you have prevented it?
10. What do you take away from this situation? What did you learn from it?
11. Can you describe another situation, possibly from a previous job, in which your mental health played a role?
    1. [If the interviewee does not describe a positive experience:] Can you describe a work situation that had a positive influence on your mental health?
12. Does your home situation play a role in how your work contributes to your mental well-being?
13. What do you need to feel good about yourself at work?
14. Do you have any questions or is there anything else you would like to say on this topic that we haven't covered?

- Explain that the interviewee will receive a follow-up email (including the signed informed consent and information letter) inviting them to evaluate the interview and share information that comes to mind after the interview.
