# Supplementary material for: A qualitative study of young workers’ experience of the psychosocial work environment and how this affects their mental health
Source: BMC Public Health. 2024 Nov 29;24:3341. doi: 10.1186/s12889-024-20760-x (PMC11607927; doi:10.1186/s12889-024-20760-x)
Supplement: Supplementary file 3 — Supplementary Material 3 [file 12889_2024_20760_MOESM3_ESM.pdf]

        - Work Condition & Experience at Work > COPSOQ. Possibilities for development (incl skill discretion) > learning new skills at work > lack of learning opportunities
    - Work Condition & Experience at Work > COPSOQ. Possibilities for development (incl skill discretion) > insufficient challenge
    - Work Condition & Experience at Work > COPSOQ. Possibilities for development (incl skill discretion) > using skills/being able to do what you are good at
- Work Condition & Experience at Work > COPSOQ. Variation of Work
    - Work Condition & Experience at Work > COPSOQ. Variation of Work > task variation
- Work Condition & Experience at Work > COPSOQ. Control over working time.
    - Work Condition & Experience at Work > COPSOQ. Control over working time. > availability outside of work hours
- Work Condition & Experience at Work > COPSOQ. Meaning of work
    - Work Condition & Experience at Work > COPSOQ. Meaning of work > affinity with service or product
    - Work Condition & Experience at Work > COPSOQ. Meaning of work > doing societally relevant work
- Work Condition & Experience at Work > COPSOQ. Predictability (receiving information to do ones work)
    - Work Condition & Experience at Work > COPSOQ. Predictability (receiving information to do ones work) > procedural clarity
- Work Condition & Experience at Work > COPSOQ. Recognition
    - Work Condition & Experience at Work > COPSOQ. Recognition > appreciation/reward
    - Work Condition & Experience at Work > COPSOQ. Recognition > not feeling seen/valued
    - Work Condition & Experience at Work > COPSOQ. Recognition > getting compliments/feeling valued
    - Work Condition & Experience at Work > COPSOQ. Recognition > trusting that one is not exploited
- Work Condition & Experience at Work > COPSOQ. Role clarity
    - Work Condition & Experience at Work > COPSOQ. Role clarity > role (un-)clarity
- Work Condition & Experience at Work > COPSOQ. Role conflicts
- Work Condition & Experience at Work > COPSOQ. Illegitimate tasks
    - Work Condition & Experience at Work > COPSOQ. Illegitimate tasks > having to do adverse job tasks
- Work Condition & Experience at Work > COPSOQ. Quality of Leadership
    - Work Condition & Experience at Work > COPSOQ. Quality of Leadership > attention for personal development
- Work Condition & Experience at Work > COPSOQ. Social Support from Colleagues
    - Work Condition & Experience at Work > COPSOQ. Social Support from Colleagues > not feeling connected to colleagues
    - Work Condition & Experience at Work > COPSOQ. Social Support from Colleagues > colleagues being approachable
- Work Condition & Experience at Work > COPSOQ. Social Support from Supervisor
- Work Condition & Experience at Work > COPSOQ. Sense of community at work
    - Work Condition & Experience at Work > COPSOQ. Sense of community at work > conviviality ("gezelligheid")
    - Work Condition & Experience at Work > COPSOQ. Sense of community at work > good colleagues
    - Work Condition & Experience at Work > COPSOQ. Sense of community at work > feeling of belonging to a team
    - Work Condition & Experience at Work > COPSOQ. Sense of community at work > talking to colleagues about non-work topics
    - Work Condition & Experience at Work > COPSOQ. Sense of community at work > colleagues working by themselves ("on an island")
    - Work Condition & Experience at Work > COPSOQ. Sense of community at work > caring colleagues
- Work Condition & Experience at Work > COPSOQ. Commitment to the workplace
- Work Condition & Experience at Work > COPSOQ. Work Engagement
- Work Condition & Experience at Work > COPSOQ. Job Insecurity
    - Work Condition & Experience at Work > COPSOQ. Job Insecurity > temporary contract
- Work Condition & Experience at Work > COPSOQ. Insecurity over working conditions
- Work Condition & Experience at Work > COPSOQ. Quality of Work
    - Work Condition & Experience at Work > COPSOQ. Quality of Work > senior colleagues conserving their ways
    - Work Condition & Experience at Work > COPSOQ. Quality of Work > effect of age and tenure
- Work Condition & Experience at Work > COPSOQ. Job Satisfaction (incl salary)
    - Work Condition & Experience at Work > COPSOQ. Job Satisfaction (incl salary) > satisfaction with salary
    - Work Condition & Experience at Work > COPSOQ. Job Satisfaction (incl salary) > role of salary
- Work Condition & Experience at Work > Work Life Conflict
- Work Condition & Experience at Work > COPSOQ. Vertical Trust
    - Work Condition & Experience at Work > COPSOQ. Vertical Trust > perceived mistrust in one's work by supervisor
    - Work Condition & Experience at Work > COPSOQ. Vertical Trust > feeling that others trust in one's work
- Work Condition & Experience at Work > COPSOQ. Horizontal trust.
- Work Condition & Experience at Work > COPSOQ. Organizational Justice
    - Work Condition & Experience at Work > COPSOQ. Organizational Justice > sharing successes
    - Work Condition & Experience at Work > COPSOQ. Organizational Justice > fair distribution of work
- Work Condition & Experience at Work > Cognitive demands (different to COPSOQ Cog Dem)
- Work Condition & Experience at Work > role of migration background
- Work Condition & Experience at Work > difficult to say "no" to requests
- Work Condition & Experience at Work > colleagues not following up on what was discussed
- Work Condition & Experience at Work > more self-esteem at work because of more work experience
- Work Condition & Experience at Work > publication pressure
- Work Condition & Experience at Work > expectations coming from colleagues
- Work Condition & Experience at Work > travelling to work

- Work Condition & Experience at Work > feeling to junior to address issues
- Work Condition & Experience at Work > role of gender
- Work Condition & Experience at Work > first work experience perceived as/becoming reference "normal"
- Work Condition & Experience at Work > colleagues having mental health issues
- Work Condition & Experience at Work > putting less effort in work because of work dissatisfaction
- Work Condition & Experience at Work > competition with other research institutes
- Work Condition & Experience at Work > overall good experience
- Work Condition & Experience at Work > colleagues suggesting to stick to regular working hours
- Work Condition & Experience at Work > depending on others to do one's work
- Work Condition & Experience at Work > experiences at work shared by colleagues
- Work Condition & Experience at Work > hierarchy in an organization
- Work Condition & Experience at Work > interruptions and distractions
- Work Condition & Experience at Work > group norms
  - Work Condition & Experience at Work > group norms > atmosphere at work
- Work Condition & Experience at Work > having young colleagues to talk to
- Work Condition & Experience at Work > discussing age-based differences at work
- Work Condition & Experience at Work > negative situation is temporary
- Work Condition & Experience at Work > not feeling connected to supervisor
- Work Condition & Experience at Work > overall bad experience at work
- Work Condition & Experience at Work > being challenged in a positive way
  - Work Condition & Experience at Work > being challenged in a positive way > knowing how to fill one's work day
    - Work Condition & Experience at Work > being challenged in a positive way > knowing how to fill one's work day > too little work
  - Work Condition & Experience at Work > being challenged in a positive way > boredom
- Work Condition & Experience at Work > employer facilitating following formal education
- Work Condition & Experience at Work > participation in decision-making processes
- Work Condition & Experience at Work > showing respect among colleagues
- Work Condition & Experience at Work > anticipating reaction of colleagues when communicating at work
- Work Condition & Experience at Work > getting negative feedback
- Work Condition & Experience at Work > reflection on own performance
- Work Condition & Experience at Work > colleagues depending on one's work
- Work Condition & Experience at Work > helping colleagues
- Work Condition & Experience at Work > feeling misunderstood
- Work Condition & Experience at Work > person-job fit
- Work Condition & Experience at Work > enjoyable work
- Work Condition & Experience at Work > physical safety (incl. aggression)
- Work Condition & Experience at Work > poor communication
- Work Condition & Experience at Work > contrasts with colleagues' experiences
- Work Condition & Experience at Work > Expectations from employer
- Work Condition & Experience at Work > expectation from employer concerning development opportunitites
- Work Condition & Experience at Work > social interactions
  - Work Condition & Experience at Work > social interactions > with non-professionals (e.g. patients, students...)
  - Work Condition & Experience at Work > social interactions > with non-colleague professionals (B2B-contacts)
  - Work Condition & Experience at Work > social interactions > with colleagues
  - Work Condition & Experience at Work > social interactions > Teamwork
- Work Condition & Experience at Work > Conflict with Supervisor
- Work Condition & Experience at Work > asking for help/support & sparren
- Work Condition & Experience at Work > (lack of) supervision/guidance
- Work Condition & Experience at Work > (lack of) Decision latitude or -authority & Autonomy
- Work Condition & Experience at Work > Conflicting task demands
- Work Condition & Experience at Work > Employer driven social activities
- Work Condition & Experience at Work > Motivation/justification for excessive workload
- Work Condition & Experience at Work > Open atmosphere to discuss job tasks
- Work Condition & Experience at Work > Pressure from supervisor
- Work Condition & Experience at Work > Working hours
- Work Condition & Experience at Work > Job crafting
- Work Condition & Experience at Work > Expectations about job
- Work Condition & Experience at Work > Development opportunities
- Work Condition & Experience at Work > Responsibilities
- Work Condition & Experience at Work > Contrast with previous job
- Work Condition & Experience at Work > Initial experience
- Work Condition & Experience at Work > Relation between function title and actual work
- Work Condition & Experience at Work > Terms of employment

	Work Condition & Experience at Work > seeing tangible results of own work	
Function and Job Task Description		
	Function and Job Task Description > salary	
	Function and Job Task Description > Type of company	
	Function and Job Task Description > Contract Hours	
	Function and Job Task Description > Sector	
	Function and Job Task Description > Supervisor/Line-Manager	
Career		
	Career > motivation to not follow-up/explore job offer	
	Career > differences in work between countries	
	Career > doing PhD does not feel like a "real" job	
	Career > difference side job and "real job"	
	Career > motivation for career change	
	Career > multiple job holding	
	Career > "real" job/work	
	Career > Still studying	
	Career > Carreer timeline	
	Career > thinking about switching job	
	Career > In between job	
	Career > Motivation to start a particular job	
	Career > Starting Education after having worked	
	Career > Relation & Combination Education and Work	
	Career > Side Jobs	
	Career > Beginning of work life	
	Career > First Job	
	Career > Current Job	
	Career > Second Job	
	Career > Transition from side job to first job	
	Career > Transition to other job	
Personal characteristic		
	Personal characteristic > competitive athlete	
	Personal characteristic > romantic relationship	
	Personal characteristic > financial situation	
	Personal characteristic > Age	
	Personal characteristic > Person is currently working	
	Personal characteristic > Personal experience	
	Personal characteristic > Personality Characteristics	
		Personal characteristic > Personality Characteristics > high sense of responsibility
		Personal characteristic > Personality Characteristics > being helpful / wanting to help others
		Personal characteristic > Personality Characteristics > fear of failure
		Personal characteristic > Personality Characteristics > being perfectionist
		Personal characteristic > Personality Characteristics > reflection on sepaerating free time and work time
		Personal characteristic > Personality Characteristics > feeling like not working enough
		Personal characteristic > Personality Characteristics > Expectation about life
Importance of work in life		
	Importance of work in life > work means regular social contact	
	Importance of work in life > parents as role model	
	Importance of work in life > loyalty for employer	
	Importance of work in life > moving for work	
	Importance of work in life > works gives structure	
	Importance of work in life > Change in importance	
Education		
	Education > Location University of Applied Sciences	
	Education > University of Applied Sciences	
